# Dynamic Colonization of *Klebsiella pneumoniae* Isolates in Gastrointestinal Tract of Intensive Care Patients

**DOI:** 10.3389/fmicb.2019.00230

**Published:** 2019-02-11

**Authors:** Qiao-ling Sun, Danxia Gu, Qi Wang, Yanyan Hu, Lingbin Shu, Jie Hu, Rong Zhang, Gong-Xiang Chen

**Affiliations:** ^1^Department of Clinical Laboratory, Second Affiliated Hospital of Zhejiang University, School of Medicine, Hangzhou, China; ^2^Key Laboratory of Tumor Molecular Diagnosis and Individualized Medicine of Zhejiang Province, Clinical Research Institute, Zhejiang Provincial People’s Hospital, People’s Hospital of Hangzhou Medical College, Hangzhou, China

**Keywords:** CRKP, KPC-2, gastrointestinal carriage, dynamical colonization, multiple clones

## Abstract

Gastrointestinal carriage is regarded as a major reservoir of *K. pneumoniae* infections, especially in intensive care patients. A total of 101 (95.3%) KPC-producing carbapenem-resistant *K. pneumoniae* (CRKP) isolates were identified among 106 CRKP isolates collected from stool samples of inpatients performing active rectal screening for carbapenem-resistant Enterobacteriaceae during hospitalization in the ICUs of a tertiary hospital between 2016 and 2017. Among them, six KPC-producing CRKP isolates from three patients (two isolates for each patient) were identified with distinct antibacterial susceptibility. Our findings showed that: (1) *bla*_KPC–2_ gene is predominant in CRKP strains isolated from the intensive care patients and can be incorporated into various plasmids that are transmissible among multiple bacterial hosts in the human gastrointestinal tract; (2) the human gastrointestinal tract has a capacity to dynamically colonize multiple clones of CRKP strains with varied plasmids, diverse antimicrobial resistance genes and virulence genes. *K. pneumoniae* colonization is an important step in progression to extraintestinal infection, which provides the rationale for establishing intervention measures to prevent subsequent infection. Thus, close surveillance on CRKP colonization, together with effective infection prevention and control measures, should be put into practice.

## Introduction

*Klebsiella pneumoniae* is a major opportunistic pathogen that can cause invasive hospital-acquired infections among immune-compromised patients especially for those from ICU ward with critical illness. Carbapenem is a first line therapy for the treatment of infections caused by multidrug-resistant *K. pneumoniae*. However, carbapenem-resistant *K. pneumoniae* (CRKP) has emerged as a public threat to cause serious infections with high mortality up to 33.24–50.06% (Xu et al., 2017). Recent data from the CHINET surveillance program showed that the prevalence of CRKP had dramatically increased from 3 to 17.9% since 2005 (Hu et al., 2016, 2017). A previous study on nationwide surveillance of clinical carbapenem-resistant Enterobacteriaceae (CRE) strains has revealed that *bla*_KPC-2_ was the most prevalent genotype in China, accounting for 73% of CRKP isolates (Zhang et al., 2017).

Carbapenem-resistant Hypervirulent *K. pneumoniae* (CR-HvKP), especially ST11 KPC-producing strains, have been increasingly reported in recent years (Zhang R. et al., 2015; Zhang Y. et al., 2015; Gu et al., 2018). They often cause invasive, even life-threatening infections with high mortality among young and healthy populations. In a previous study, we reported an outbreak of ST11 CR-HvKP in the ICU ward; further study indicated that the acquisition of a virulence plasmid carrying *rmpA2* and aerobactin biosynthesis genes by classic ST11 CRKP strains played a critical role (Gu et al., 2018).

Gastrointestinal carriage has been regarded as a major reservoir of *K. pneumoniae* infections, especially in intensive care patients (Gorrie et al., 2017). A prospective study in 1971 indicated that 18.5% patients colonized with multidrug-resistant *K. pneumoniae* after hospital admission had higher risk to develop subsequent infection caused by identical bacteria within 21 days compared to those who did not become intestinal carriers (45% vs. 11%) (Martin and Bachman, 2018). A 2016 study reported similar colonization rates (23%) and increased risk of infection following colonization (5.2% in colonized vs. 1.3% in noncolonized) (Martin et al., 2016). The carriage rate of CRKP is reported to range from 8 to 9% in medical and surgical departments to 5% in intensive care units (Wiener-Well et al., 2010). CRKP colonization can persist and spread silently for years, even trigger a clonal outbreak in long-term care facilities while newly colonized patients can develop fatal infections (Martin and Bachman, 2018). Our previous study showed the evolution of tigecycline- and colistin-resistant CRKP has occurred *in vivo* under the antibiotic selection and CRKP could persistently colonize in the human gastrointestinal tract for 3 years even without antibiotic selection pressure (Zhang et al., 2018). Previous reports mainly focused on epidemiological data of CRKP carriage and associated risk factors (Papadimitriou-Olivgeris et al., 2012; Feldman et al., 2013; Papadimitriou-Olivgeris et al., 2013), in this study, we are going to investigate the molecular characterization of multiple clones of CRKP strains colonized in the gastrointestinal tract of intensive care inpatients, aiming at illustrating certain discipline underlying the colonization of CRKP.

## Materials and Methods

### The Rectal CRKP Isolates From the Intensive Care Inpatients

A total of 106 *K. pneumoniae* isolates that exhibited carbapenem resistance phenotype (MIC value of meropenem ≥4 μg/ml) were identified from stool samples of inpatients performing active rectal screening for CRE during hospitalization in the ICUs of Second Affiliated Hospital of Zhejiang University, School of Medicine (Hangzhou, China) between 2016 and 2017. Identification of species was confirmed via a matrix-assisted laser desorption/ionization time-of-flight mass spectrometry (Bruker Daltonik GmbH, Bremen, Germany). The presence of carbapenem-resistant genes (*bla*_NDM_, *bla*_KPC_, *bla*_IMP_, *bla*_V IM_, and *bla*_OXA–48_) was screened using PCR (Zhang et al., 2017). Positive products were validated with Sanger DNA sequencing. A total of 101 (95.3%) KPC-2-producing CRKP isolates were identified among 106 rectal CRKP isolates. Among them, six KPC-2-producing CRKP isolates from three patients (two isolates for each patient) were identified with distinct antibacterial susceptibility. In order to investigate the origin of the intestinal CRKP isolates and further look into the relationship between the rectal colonization and extraintestinal infections, we reviewed medical history of the three inpatients in detail and subjected the KPC-2-producing CRKP isolates collected during peri-hospitalization period to phenotypic and genotypic characterization.

### Phenotypic Characterization

The antimicrobial susceptibilities of the isolates were determined using a broth microdilution procedure and the interpretations were in accordance with the guideline document M100-S26 established by Clinical and Laboratory Standards Institute (CLSI, 2016).

We did pulsed-field gel electrophoresis (PFGE), S1-PFGE, and Southern hybridization as previously reported (Huang et al., 2016). A dendrogram was generated from the homology matrix with a coefficient of 0.5% using the unweighted pair-group method using arithmetic averages (“UPGMA”) to describe the relationships among PFGE profiles. Isolates were considered to belong to the same PFGE group if their Dice similarity index was ≥85%.

The virulence gene *rmpA2*, encoded on the virulence plasmid, was identified by PCR as previously described (Gu et al., 2018). All isolates were performed with the string test to identify the hypermucoviscous phenotype as described previously (Gu et al., 2018). As a test of virulence, we quantified virulence with *Galleria mellonella* larva models. The *G. mellonella* larva was injected into the hemocoel of each caterpillar via the last left proleg with 10 μl suspensions of a *K. pneumoniae* strain containing a final concentration of 10^6^ CFU/mL, incubated at 37°C and observed every 12 h for 3 days. The effect of 1 × 10^6^ CFU of each *K. pneumoniae* strain on survival was assessed in *G. mellonella*. HvKP strain *K. pneumoniae* 4 and strain PC *K. pneumoniae* 4, reported in a previous study (Gu et al., 2018), were treated as controls. HvKP strain *K. pneumoniae* 4 was a ST11 KPC-producing hypermucoviscous strain harboring various virulence genes (*rmpA2*, *iutA*, *iroE*, *kpn*, *ycfM*, *iucABCD*, *mrkABCDF*, and *fimA–H*), located on the virulence plasmid pLVPK. Strain PC *K. pneumoniae* 4 is a mutant strain of *K. pneumoniae* 4, of which the virulence plasmid has been removed in plasmid curing experiments. Strain PC *K. pneumoniae* 4 was negative for string test and demonstrated reduced virulence in *G. mellonella* models. Two control groups were performed: the first group was inoculated with PBS to monitor for killing due to physical trauma and attrition while the second received no injection. Eight larvae from each group were examined and all the experiments were performed in triplicates. Kaplan-Meier survival curves were plotted using GraphPad Prism version 7.00, the log rank (Mantel-Cox) test was used to analyze whether significant differences (*P* < 0.05) in the survival rates of the infected *G. mellonella* larvae were observed.

### Whole Genome Sequencing and Bioinformatics Analysis

Genomic DNA was extracted from overnight cultures by using the PureLink Genomic DNA Mini Kit (Invitrogen, Carlsbad, CA, United States) and was subjected to whole genome sequencing using 150 bp pair-end strategies with the Illumina HiSeq X10 platform. Raw reads were trimmed and assembled to contigs using SPAdes v3.11.1 (Bankevich et al., 2012). Assembled genome sequences were submitted to the NCBI database with accession number QMKA00000000, QMKB00000000, QMKC00000000, QMKD00000000, QMKE00000000, and QMKF00000000. Genome sequences were annotated with the RAST tool (Overbeek et al., 2013) and Prokka (Seemann, 2014). Multilocus sequence types (MLSTs), virulence-associated genes encoding yersiniabactin, aerobactin, salmochelin and the regulators of mucoid phenotype were determined with Kleborate (Pichler et al., 2017). Serotyping was performed using Kaptive (Wyres et al., 2016). Acquired antibiotic resistance genes were identified with ResFinder 2.1 (Zankari et al., 2012). Heatmap of the antimicrobial resistance genes was generated using an in-house script.

### Ethics Statement

The study was approved by the Ethics Committee of Second Affiliated Hospital of Zhejiang University, School of Medicine (2017-099). All subjects gave written informed consent in accordance with the Declaration of Helsinki.

### Biosafety Statement

All concerns related to the safe and appropriate use of human-derived materials, infectious agents, or genetically modified organisms were approved by the Institutional Biosafety Committee of Second Affiliated Hospital of Zhejiang University, School of Medicine. All experiments were conducted under the guidelines from the Biological Agent Reference Sheet.

## Results

**FIGURE 1:**
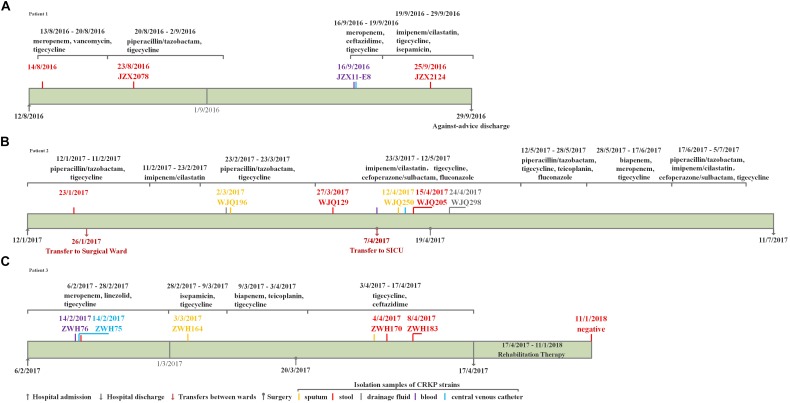
The clinical course, therapeutic regimen, and outcomes of Patient 1 **(A)**, Patient 2 **(B)** and Patient 3 **(C)** who carried CRKP isolates.

**FIGURE 2:**
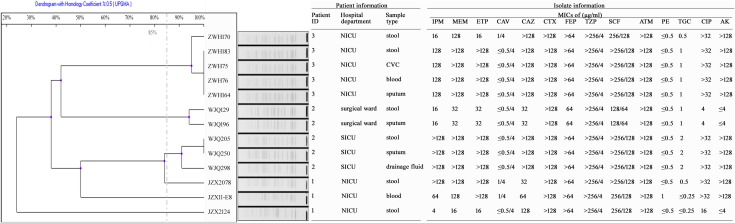
Antimicrobial susceptibility and PFGE profiles of 13 CRKP isolates collected from three inpatients. IPM, Imipenem; MEM, Meropenem; ETP, Ertapenem; CAV, Ceftazidime-avibactam; CAZ, Ceftazidime; CTX, Cefotaxime; FEP, Cefepime; TZP, Piperacillin-tazobactam; SCF, Cefoperazone-sulbactam; ATM, Aztreonam; PE, colistin; TGC, Tigecycline; CIP, Ciprofloxacin; AK, Amikacin; CVC, central venous catheter.

Three elderly patients aged 61–70 years were admitted to the ICU between August 2016 and February 2017. The three patients all developed diseases including bacterial pneumonia and bloodstream infections and underwent surgery, followed by antimicrobial treatment and mechanical ventilation, but they responded differently to antibiotic treatment. Only two patients, Patient 2 and 3, succeeded in infection control while Patient 1 experienced a persistent fever and pulmonary infection till against-advice discharge. Detailed information about the patients is available in the Figure 1, 2. Active rectal screening for CRE was conducted for all the patients during hospital admission and peri-hospitalization period, and all the three patients were found to carry the KPC-producing CRKP strains in the gastrointestinal tract on admission and during stays at healthcare settings. Thereinto, two CRKP isolates from stool samples of each patient presented distinct antimicrobial susceptibility profiles. In order to study the origin of the intestinal CRKP carriage and the relationship between the rectal colonization and extraintestinal infections, three, five, and five more KPC-producing CRKP isolates from various types of specimens including stool were successively identified in Patient 1, Patient 2, and Patient 3 during peri-hospitalization period, respectively (Figure 1, 2).

### Acquisition of KPC-Producing CRKP Isolates From Stool Samples

**FIGURE 3:**
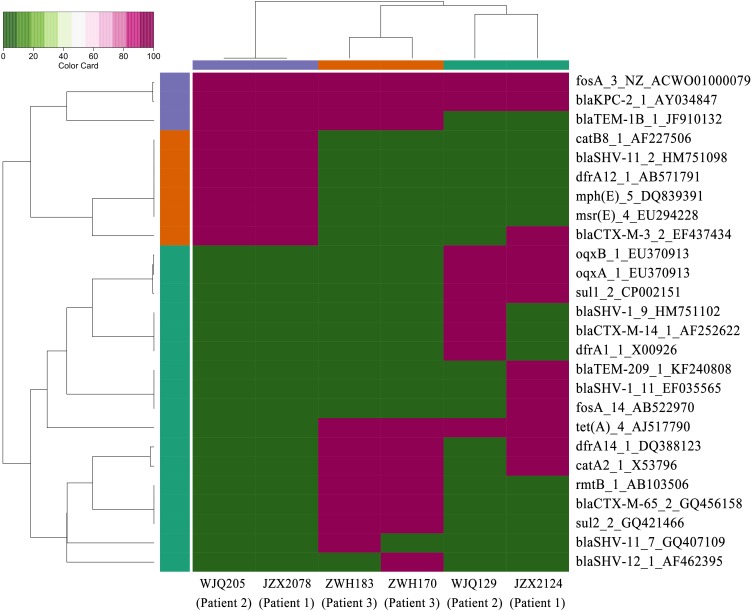
Heatmap of antimicrobial resistance genes in the CRKP isolates from stool samples. Red and green boxes indicate the presence and absence of the corresponding antimicrobial resistance genes, respectively.

Patient 1, a 71-year old woman, had previously been admitted to a local hospital for acute ventricular hemorrhage and was transferred to the Neurological intensive care unit (NICU) of our hospital on Aug 12, 2016. Three KPC-2-producing CRKP isolates (namely JZX2078, JZX2124, and JZX11-E8) were isolated. Isolate JZX2078 was recovered from stool sample after 1 week of admission, belonging to ST11 with unidentified serotype. It harbored three plasmids with sizes of ∼230, ∼210, and ∼80 Kb. The ∼210 Kb plasmid was a pLVPK-like virulence plasmid which bears *iucABCD*, *iroBCD*, and *rmpA2* genes. The ∼230 Kb plasmid was a pKP04VIM (KU318421.1)-like plasmid, while the ∼80 Kb one was a *bla*_KPC–2_-bearing p69-2 (CP025458.1)-like plasmid (Table 1). The second isolate JZX2124 was also isolated from stool a week before discharge. It was identified as a K19, ST1 strain which harbored two multidrug resistance plasmids, the ∼130 Kb p11219-CTXM (MF133442.1)-like plasmid and the ∼170 Kb, *bla*_KPC-2_-bearing pIT-06C07 (LT009688)-like plasmid (Table 1). Compared to isolate JZX2078 with 9 antibiotic resistance genes, isolate JZX2124 carried 12 resistance genes including *tet(A), oqxAB* genes and two different genes encoded for extended spectrum β-lactamases (ESBLs) as shown in Figure 3. And the third isolate JZX11-E8 was recovered from blood. With completely different antibacterial susceptibility profiles and distinct PFGE patterns (Figure 2), these three isolates were proved to originate from different clones.

**Table 1:** Sequence information for rectal CRKP isolates from the three inpatients.

Strain	Patient ID	Number of contigs >500 bp	Sequence length (bp)	G+C (%)	N50	MLST	Aerobactin	Salmochelin	Hypermucoidy	Serotype
JZX2078	1	139	5863800	56.78	151743	ST11	*iucABCD*	*iroBCD*	*rmpA2*	unidentified
JZX2124	1	425	6015115	56.7	202420	ST1	–	–	–	K19
WJQ129	2	149	5479176	57.13	217169	ST290	–	–	–	K21
WJQ205	2	231	5948640	56.71	99385	ST11	*iucABCD*	*iroBCD*	*rmpA2*	K64
ZWH170	3	159	5736283	57.02	129409	ST11	–	–	*rmpA*	K64
ZWH183	3	219	5852344	57.04	99385	ST11	*iucABCD*	–	*rmpA*, *rmpA2*	K64

Patient 2, a 66-year old man, was admitted to the Surgical Intensive Care Unit (SICU) for severe acute pancreatitis with acute respiratory distress syndrome on January 12th, 2017. Two weeks after admission, he was transferred to Surgical Ward preparing for a surgery of cholecystectomy and peritoneal lavage and drainage, immediately followed by transferring to the SICU and experiencing one more surgery of intraperitoneal hemostasis and peritoneal lavage and drainage. Two CRKP isolates, WJQ129 and WJQ196, were isolated from stool and sputum samples, respectively, during his staying in Surgical Ward, while three CRKP isolates (WJQ205, WJQ250, and WJQ298) were collected from stool, sputum, and drainage fluid samples, respectively, during his staying in SICU. With similar antimicrobial susceptibility profiles and PFGE patterns, isolates WJQ129 and WJQ196 shared high homology. The same situation was presented for isolates WJQ205, WJQ250, and WJQ298. However, as shown in Figure 2, isolates recovered from the patient during his earlier staying in Surgical Ward were completely distinct from those recovered during his later staying in SICU. It may be suggested that Patient 2 was successively infected by two different strains of KPC-producing *K. pneumoniae* isolated from different ICU wards. CRKP colonized in the gastrointestinal tract would easily cause extraintestinal infections once host defense system is weakened. Isolate WJQ129 was an ST290, K21 strain which harbored 9 antibiotic resistance genes (Table 1, Figure 3) and two plasmids with sizes of ∼230 and ∼90 Kb. The ∼230 Kb *K. pneumoniae* strain FDAARGOS_443 plasmid unnamed1 (CP023937.1)-like plasmid carries resistance genes *tet(A)*, *qnrS*, *bla*_CTX–M–14_, and *dfrA1*. The ∼90 Kb p628-KPC (KP987218.1)-like plasmid carries *bla*_KPC–2_. Isolate WJQ205 belonged to ST11, and identified as K64, carrying three plasmids (∼240, ∼210, and ∼80 Kb) (Table 1). The ∼210 Kb plasmid is a pLVPK-like virulence plasmid, carrying *iucABCD*, *iroBCD*, and *rmpA2* genes. The ∼240 and ∼80 Kb plasmids are pA324-IMP (MF344566)- and *bla*_KPC–2_-bearing p69-2 (CP025458.1)-like plasmids, respectively.

Patient 3, a 61-year old woman, was admitted to our NICU for disorder of consciousness after a surgery of eliminating intracranial hematoma and bilateral external ventricular drainage in a local hospital. Five CRKP isolates were recovered from different samples belonging to two highly similar PFGE patterns. Interestingly, two homologous CRKP clones ZWH170 and ZWH183, collected from stool samples at short intervals, were both ST11, K64 serotype and harbored highly similar resistance genes, but showed different antibacterial susceptibility profiles (Table 1 and Figure 2, 3). Isolate ZWH170 carried plasmids with sizes of ∼138 and ∼78 Kb, while isolate ZWH183 carried one more plasmid which is around ∼105 Kb. The ∼138 Kb plasmid was a pKPC-CR-HvKP4-like resistance plasmid carrying *bla*_KPC–2_ and *bla*_CTX–M–65_ genes. The ∼78 Kb plasmid was a p675920-2-like multidrug resistance plasmid carrying *bla*_LAP–2_, *qnrS1*, *tet(A)*, and *sul2* genes. Additionally, the ∼105 Kb plasmid was a pLVPK-like virulence plasmid. After half-year rehabilitation therapy in another hospital, the patient agreed to provide the stool sample for follow-up investigation. Surprisingly, CRKP was no longer detected from her stool samples.

### Characterization of Virulence in CRKP Isolates

**FIGURE 4:**
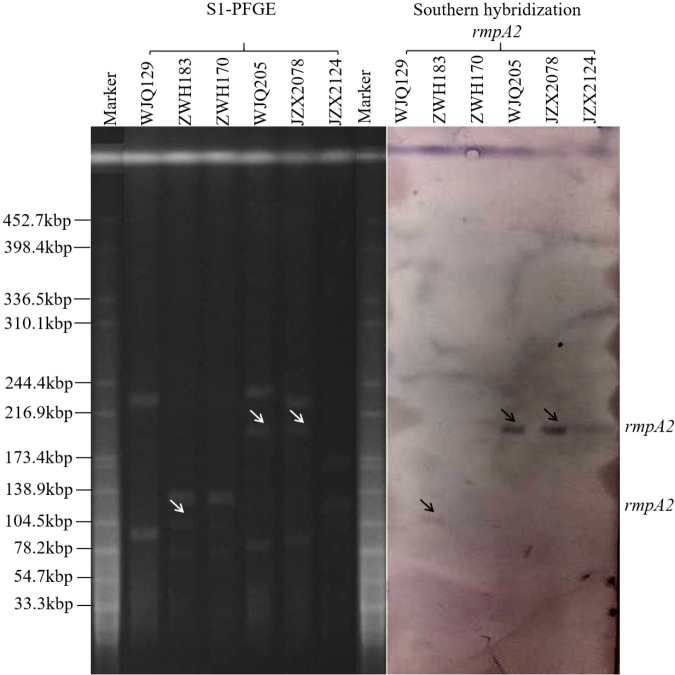
Results of S1-PFGE and Southern hybridization of marker gene of the virulence plasmid *rmpA2* harbored by KPC-2-producing CRKP isolates recovered from stool samples of three patients.

Six KPC-producing *K. pneumoniae* isolates (JZX2078, JZX2124, WJQ129, WJQ205, ZWH170, and ZWH183) from stool samples of three patients were selected for further virulence characterization. According to the presence of virulence plasmid, the six isolates were divided into two groups: *rmpA2*-positive strains (JZX2078, WJQ205, and ZWH183) and *rmpA2*-negative strains (JZX2124, WJQ129, and ZWH170). S1-PFGE and Southern hybridization of the marker gene of the virulence plasmid *rmpA2*, which was hybridized to the roughly 210 Kb and 105 Kb plasmids, confirmed the presence of the virulence plasmid in three of the ST11 KPC-producing CRKP isolates (JZX2078, WJQ205, and ZWH183). The ∼210 Kb pLVPK-like virulence plasmid, harboring *rmpA2*, *iroBCD*, and *iucABCD* genes, were identified in two ST11 CRKP strains isolated from different ICU wards. The ∼105 Kb virulence plasmid in isolate ZWH183, carrying the *rmpA2*, *iucABCD* genes was absent in isolate ZWH170, suggesting the attenuated virulence potential. The results of S1-PFGE and Southern hybridization were described in Figure 4.

**FIGURE 5:**
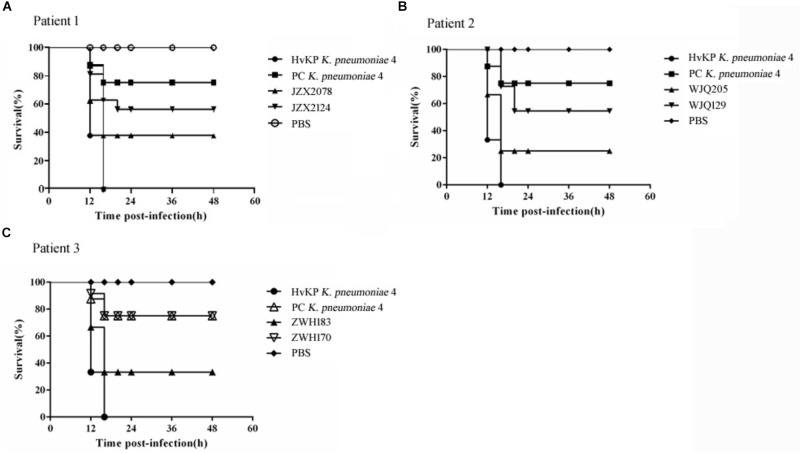
Virulence potential of KPC-2-producing CRKP isolates from Patient 1 **(A)**, Patient 2 **(B)** and Patient 3 **(C)** in the *G. mellonella* infection models. The effect of 1 × 10^6^ CFU of each *K. pneumoniae* strain on survival was assessed in *G. mellonella*. HvKP strain *K. pneumoniae* 4 was a ST11 KPC-producing hypermucoviscous strain harboring various virulence genes, located on the virulence plasmid pLVPK. Strain PC *K. pneumoniae* 4 is a mutant strain of *K. pneumoniae* 4, of which the virulence plasmid has been removed in plasmid curing experiments. Strain PC *K. pneumoniae* 4 was negative for string test and demonstrated reduced virulence in *G. mellonella* models.

All the six CRKP isolates from stool samples were negative for string test. Moreover, in *G. mellonella* larva models, the survival rate of larva infected with *rmpA2*-carrying CRKP strains (JZX2078, WJQ205, and ZWH183) ranged from 20 to 40% at 16 h after infection; as for *rmpA2*-negative CRKP strains (JZX2124, WJQ129, and ZWH170), 60–80% of the larva survived at 20 h (Figure 5). The control group infected with HvKP strain *K. pneumoniae* 4 resulted in 0% survival by 16 h, whilst 70% survival was reached in larva infected with PC *K. pneumoniae* 4 after 16 h. The survival rate of *G. mellonella* larvae infected with *rmpA2*-carrying CRKP strains (WJQ205 and ZHW183) was significantly lower than that infected with the counterpart *rmpA2*-negative CRKP strains (WJQ129 and ZWH170) (*P* < 0.05). However, there was no difference on the survival rate of *G. mellonella* larvae between the CRKP strains JZX2078 and JZX2124 (*P* > 0.05). The survival rate of *G. mellonella* larvae infected with *rmpA2*-carrying CRKP strains (WJQ205, ZHW183, and JZX2078) and *rmpA2*-negative CRKP strains (JZX2124, WJQ129, and ZWH170) showed statistical significance with that infected with HvKP (*P* < 0.05). Interestingly, the survival rate of *G. mellonella* larvae infected with all the *rmpA2*-carrying CRKP strains was significantly lower than that PC *K. pneumoniae* 4 (*P* < 0.05), while *rmpA2*-negative CRKP strains all exhibited no difference on the survival rate of *G. mellonella* larvae with PC *K. pneumoniae* 4 (*P* > 0.05).

## Discussion

Gastrointestinal colonization is regarded as a common and significant reservoir of *K. pneumoniae* in terms of risk of transmission and infection (Martin and Bachman, 2018). Previous studies have found that *K. pneumoniae* gastrointestinal colonization was significantly associated with subsequent infections in inpatients (odds ratio >4), and 5% of colonized patients developed infections. In addition, 80% concordance was showed between infecting and colonizing isolates of *K. pneumoniae* within infected patients (Martin et al., 2016; Gorrie et al., 2017; Martin and Bachman, 2018). In our study, each of Patient 2 and Patient 3 had a homologous strain in the gut and an invasive site according to the Figure 2, suggesting that CRKP in the gastrointestinal tract would readily cause extraintestinal infections once the host defense system is weakened. Thus, CRKP colonization in the gastrointestinal tract is of significant importance.

As was widely reported (Holt et al., 2015; Lam et al., 2018), *K. pneumoniae* isolates are often volatile with a wide spectrum of diversity. The human gut is always considered as a reservoir for antibiotic resistance genes, with various species and abundant genes; horizontal transfer of resistance genes is extremely active. CRKP isolates colonized in the gut can be even more diversified. In the current study, three KPC-producing CRKP isolates from Patient 1 were highly heterogeneous belonging to different clones. Interestingly, the two from her intestine demonstrated completely different antibacterial susceptibility, PFGE and plasmid profiles. Patient 2 was successively infected by two different strains of CRKP isolated from different ICU wards, which indicated that hospital-acquired CRKP strains among the ICU ward is responsible for the colonizing and infecting strains in inpatients, and multiple clones of CRKP strains might spread in the clinical setting. *bla*_KPC–2_ gene was the predominant genotype in 95.3% CRKP isolates and two *bla*_KPC–2_-bearing plasmids belonged to two major types of ∼138 Kb and ∼80 Kb in size, suggesting that the *bla*_KPC–2_ gene can be incorporated into various extrachromosomal elements which are capable of horizontal transfer among multiple bacterial hosts in the human gastrointestinal tract.

According to a previous report (Feldman et al., 2013), 74% of the patients were identified with gastrointestinal carriage of CRKP 30 days after discharge from hospital, and when it came to 6 months, the proportion declined to <30%. Persistent carriage of CRKP was associated with several risk factors, including catheter use, long-term care facilities, recent acquisition (<4 months), and a low functional status (Feldman et al., 2013). Additionally, a hypothesis was posed that CRKP could colonize in host cells to circumvent phagocytosis by immune cells, and survival of CRKP within host cells might serve as a reservoir to protect from antibiotic treatments and enable long-term coexistence with the host (Yang et al., 2018). In this study, all the three inpatients underwent mechanical ventilation, carried CRKP isolates in 2 weeks after hospital admission and had suffered from bloodstream infection caused by CRKP strains during hospitalization. The persistent carriage of CRKP in gastrointestinal tract could last for 1–6 months in our study, inferring that long-term hospital stay and prolonged antibiotic usage could acquire CRKP strains from the settings and enrich them in the host gastrointestinal tract, and CRKP might survive intracellularly to persist in the gastrointestinal colonization. Interestingly, after the half-year rehabilitation therapy in another hospital, CRKP was no longer detected from the stool samples of the Patient 3.

ST11 has been one of the most prevalent MLST in the clinical CRKP strains among different parts of the world. Previous study reported an outbreak of ST11 CR-HvKP in the ICU of our hospital, whose emergence was the result of acquisition of a ∼170 Kb virulence plasmid pLVPK carrying *rmpA2*, *iroBCD*, and *iucABCD* genes by classic ST11 CRKP strains (Gu et al., 2018). A ∼210 Kb pLVPK-like virulence plasmid carrying *rmpA2*, *iroBCD*, and *iucABCD* genes was identified in two ST11 KPC-producing CRKP isolates in the present study, which expressed lower virulence compared to that of the ST11 CR-HvKP previously described but higher virulence than that of the CRKP isolates without virulence plasmids. Notably, a comparison of the two homologous isolates ZHW170 and ZHW183 revealed that ZWH183 carried an additional virulence plasmid (∼105 Kb, co-carrying the *rmpA2* and *iucABCD* genes), which may be a glimpse of dynamic exchange of mobile elements in the gastrointestinal tract. Moreover, as was previously reported, CR-HvKP was found to account for only 3% of infections caused by the ST11 CRKP strains across China (Gu et al., 2018). Consistently, most of CRKP isolates in this study are identified with low virulence. *K. pneumoniae* has a wider ecological distribution, significantly more varied DNA composition, greater antimicrobial resistance gene diversity and a higher plasmid load than other Gram negative opportunists, which means more opportunity to survive within and transfer between multiple environmental and animal-associated hosts; to capture plasmids from environmental microbial communities; to maintain antimicrobial resistance gene-carrying plasmids for long periods; and to transfer plasmids to other clinically important Gram negative bacteria (Wyres and Holt, 2018). Our finding showed that ST11 *rmpA2*-positive CRKP strains harbored three plasmids, of which two carried antimicrobial resistance genes and the third one was a virulence plasmid; *rmpA2*-negative CRKP strains only had two multidrug resistance encoding plasmids without virulence plasmid. It is suggested that the human gastrointestinal tract has a great capacity to colonize and enrich multiple clones of CRKP strains with varied plasmids as well as diverse antimicrobial resistance genes and virulence genes, which presumably due to frequent episodes of antibiotic treatment.

Colonization is believed as an important step in progression to extraintestinal infection which provides the rationale for establishing intervention measures to prevent subsequent infection by identifying the colonized patients. It is urgent matter to take the surveillance of rectal CRE carriage into routine test after admission.

## Author Contributions

QS did strain characterization and participated in manuscript writing. DG did the whole-genome sequencing and participated in manuscript writing. QW, YH, and JH participated in collecting the clinical data and strain characterization. LS did the *Galleria mellonella* infection experiments. RZ participated in the research design, data interpretation, and manuscript writing. G-XC designed and supervised the study, interpreted the data and wrote the manuscript.

## Conflict of Interest Statement

The authors declare that the research was conducted in the absence of any commercial or financial relationships that could be construed as a potential conflict of interest.
